# Ternary nanocomposite comprising MnO_2_, GQDs, and PANI as a potential structure for humidity sensing applications

**DOI:** 10.1038/s41598-023-48928-2

**Published:** 2023-12-08

**Authors:** Mohamed Morsy, Islam Gomaa, Abd Elhamid M. Abd Elhamid, Heba Shawkey, Mohamed Aly Saad Aly, Amir Elzwawy

**Affiliations:** 1 Building Physics and Environment Institute, Housing and Building National Research Center (HBRC), Dokki, Giza, 12311 Egypt; 2 Nanotechnology Research Centre (NTRC), The British University in Egypt (BUE), Suez Desert Road, El-Sherouk City, Cairo, 11837 Egypt; 3 Nanotechnology Lab., Electronics Research Institute, El Nozha, Cairo, 11843 Egypt; 4 Microelectronics department, Electronics Research Institute, El Nozha, Cairo, 11843 Egypt; 5 Department of Electrical and Computer Engineering at Georgia Tech Shenzhen Institute (GTSI), Tianjin University, Shenzhen, 518052 Guangdong China; 6 Ceramics Department, National Research Centre (NRC), 33 El-Bohouth St, Dokki, 12622 Cairo Egypt

**Keywords:** Materials science, Nanoscience and technology, Physics

## Abstract

Humidity sensing has been offering a noticeable contribution in different industrial, medical, and agricultural activities. Here, graphene quantum dots doped with polyaniline (PANI) and MnO_2_ were successfully prepared. The synthesized system is exposed to a set of structural, morphological, and optical investigations. The apparent crystallite size is less than 30 nm, reflecting the nanoscale of the structure, and thus validating the preparation route as evident on XRD pattern. SEM images show a fibrous structure where polyaniline dominates and covers most of the structure’s surface. The evident bands of the FTIR spectrum are designated to the component used in synthesis confirming the chemical structure of the fabricated system. The humidity sensing study of the synthesized structure is carried out through a wide range of relative humidity (RH) levels range of 11–97%. The response and recovery times of the fabricated structure are found to be around 120 and 220s, respectively.

## Introduction

The manipulation and monitoring of the environments surrounding humidity are demanded in various applications in recent years. Inherently, humidity sensors emerged and evolved in versatile areas such as the agricultural sector, food storage, medical devices, and others^1–10^. Optimizing the sensor performance concerning stability, detection range, sensitivity, rapid response, fast recovery elapsed durations, and cost-effectiveness is the major challenge facing the employment of humidity sensors and expanding their scope in these directions^11–15^. From this aspect, the selection of the materials and synthesis protocol involved in humidity sensors design is essential^16–19^. Numerous materials such as ceramics, metal oxide semiconductors, polymeric matrices, and carbonic substances were investigated for the use in humidity sensing devices^18,20–22^. The operation of nanocomposites as a humidity sensor is favorable due to their elevated surface-to-volume ratio and their capability to provide enhanced physicochemical specifications. Graphene quantum dots (GQDs), a member of the carbon nanomaterials family comprising graphene sheet layers with reduced lateral dimension have been incorporated in many composite materials. GQDs possess few desirable physicochemical properties such as noticeable bandgap (non-zero bandgap, dissimilar from graphene) and quantum confinement, thus, supporting potential applications in energy, medical, and sensing fields^23–25^.

MnO_2_ is a promising metal oxide owing to its chemical stability, specific capacitance, ease of acquisition, tailorable morphology, and reduced cost^26,27^. It was reported that the combination of MnO_2_ and graphene derivates hinders the stacking of the graphene layers due to the contribution of van der Waals forces. Moreover, MnO_2_ gains a better dispersion due to the presence of the graphene layers^28^.

Owing to its superior conductivity, thermal stability, and sensitivity to gases, polyaniline (PANI) became an ideal polymeric candidate^29,30^. PANI was previously reported as a promising candidate in sensing applications^18,31,32^, anti-corrosion coatings^33^ Photocatalysis^34,35^, optoelectronics^36,37^, and energy storage appliances^38–42^.

A few disadvantages are limiting the wide-spread employment of PANI and opposing its functionality and application such as insolubility in some solvents and its limited mechanical stability^43^. Characteristically, the humidity sensing devices using metal oxide constrain a few undesirable specifications such as increased lag, lowered response, and prolonged recovery and response times^44,45^.

Therefore, the combination of GQDs with other metal oxide materials to acquire a hybrid binary or ternary system facilitates novel tailored characteristics and expands the scope for enhanced functionality. Researchers devoted a desirable set of efforts and experimentation towards overcoming the aforementioned limitations of PANI through the implementation of organic–inorganic hybrid composites for the humidity sensing devices^3,46–48^. The implementation of PANI and metal oxide promotes the adsorption locations accompanied by raised thermal stability and conductivity. Furthermore, the incorporation of PANI into graphene or graphene derivatives can enhance the overall conductivity, mechanical stability, functional groups, and more.

Motivated by the aforementioned points, the ternary system composed of PANI as a polymeric matrix and GQDs with MnO_2_ metal oxide to work as a humidity sensor was investigated in this work. To the authors’ best knowledge, this work is the first to report on this structure as a humidity sensor.

Khouloud Jlassi et al., fabricated functionalized GQDs films from graphene waste using the spin coating method for humidity sensing applications. The synthesized films provided a low hysteresis (~ 2%) at a 30%RH level. Furthermore, the response and recovery times were found to be 15 s and 55 s, respectively^49^. In another report, authors synthesized silver nanoparticles doped with GQDs through the hydrothermal process. The report signifies the 1:1 ratio of Ag: GQDs to deliver an optimum response of 98% within a 25–95% humidity range at ambient temperature^50^. The following report revealed a humidity sensor formed by graphene quantum dots doped with chitosan and chemically synthesized was reported. The outcomes revealed a fast response and recovery times of 3 and 36 s, respectively, with a limited hysteresis of around 1.6%. The humidity sensing studies for PANI-containing composites was also reported^51^.

B. Chethan et al., synthesized a polyaniline-yttrium oxide composite prepared via the mechanical mixing method and studied the composite for humidity sensing applications. The structure delivered an optimum sensing response of 99% with a nominal response and recovery times of 3 and 4 s, respectively ^1^. Besides, PANI-titania nanotube-rGO was also investigated for humidity sensing within a wide humidity range of 7–97% RH. The system sensitivity of 3.28 MΩ/RH% and a rapid response of 13 s was observed^18^. Polyaniline-doped WO_3_ was prepared by in situ deposition technique as a single-step polymerization procedure. The results elucidated a linear behavior throughout a humidity range of 10 and 95% RH^52^. MnO_2_-CaO was also studied for humidity sensing and reporting an average sensitivity of ∼2.225 μW/% RH with a nominal response and recovery times of 47 and 59 s, respectively^53^.

Despite these efforts directed towards the humidity sensing area, and to the best of our knowledge, the introduction of a humidity sensor based on GQDs-PANI-MnO_2_ composite was not studied. In this report, GQDs-PANI- MnO_2_ nanocomposite via a feasible two-pot process is successfully synthesized. The structural, optical, and morphological implication of the desired structure is explored. The humidity sensing performance is introduced through a wide span of humidity levels (11–97% RH). The proposed composite is promising for humidity sensing and related applications.

## Materials and methods

### Chemicals

Potassium permanganate (KMnO_4_), Ammonium persulfate ((NH_4_)_2_S_2_O_8_) (APS), Aniline (C_6_H_5_NH_2_), hydrochloric acid (HCl, 35%), Citric acid (99.5%), and sodium hydroxide (≥ 97%) were obtained from Fisher chemical. Toluene (Toluene, 99.85%, Extra Dry, AcroSeal™) was purchased from Thermo Scientific Chemicals. The above-mentioned chemicals and reagents were used as delivered without any added purification or treatments. The deionized Milli-Q water was used during this experiment.

### Synthesis of graphene quantum dots (GQDs)

GQDs were prepared as per the previous report^54^. Citric acid was used as a carbon source to create graphene quantum dots (GQDs). In brief, 5 g of citric acid was melted at 180 °C for 12 h, 1.5 M NaOH solution was added dropwise to the melted thick citric acid solution at ambient temperature, and then hydrothermal treated at 180°C for an additional 12 h. The resulting dark brown powder was removed from the liquor using dialysis filtration, washed, and dried overnight in a vacuum oven at 70 °C.

### Synthesis of MnO_2_

To acquire the desired MnO_2_, 0.6 g of KMnO_4_ were added to 45 ml of DI water, next, 1.5 ml of sulphuric acid (H_2_SO_4_, 37%) was added under sustained magnetic stirring for an hour. To obtain the final powder, the solution was then transferred into an autoclave and kept at 120 ºC for 4 h. Afterward, the powder was purified and cleaned using water, and ethanol till acquiring the final dried MnO_2_.

### Synthesis of GQDs/MnO_2_/PANI

**Figure 1:**
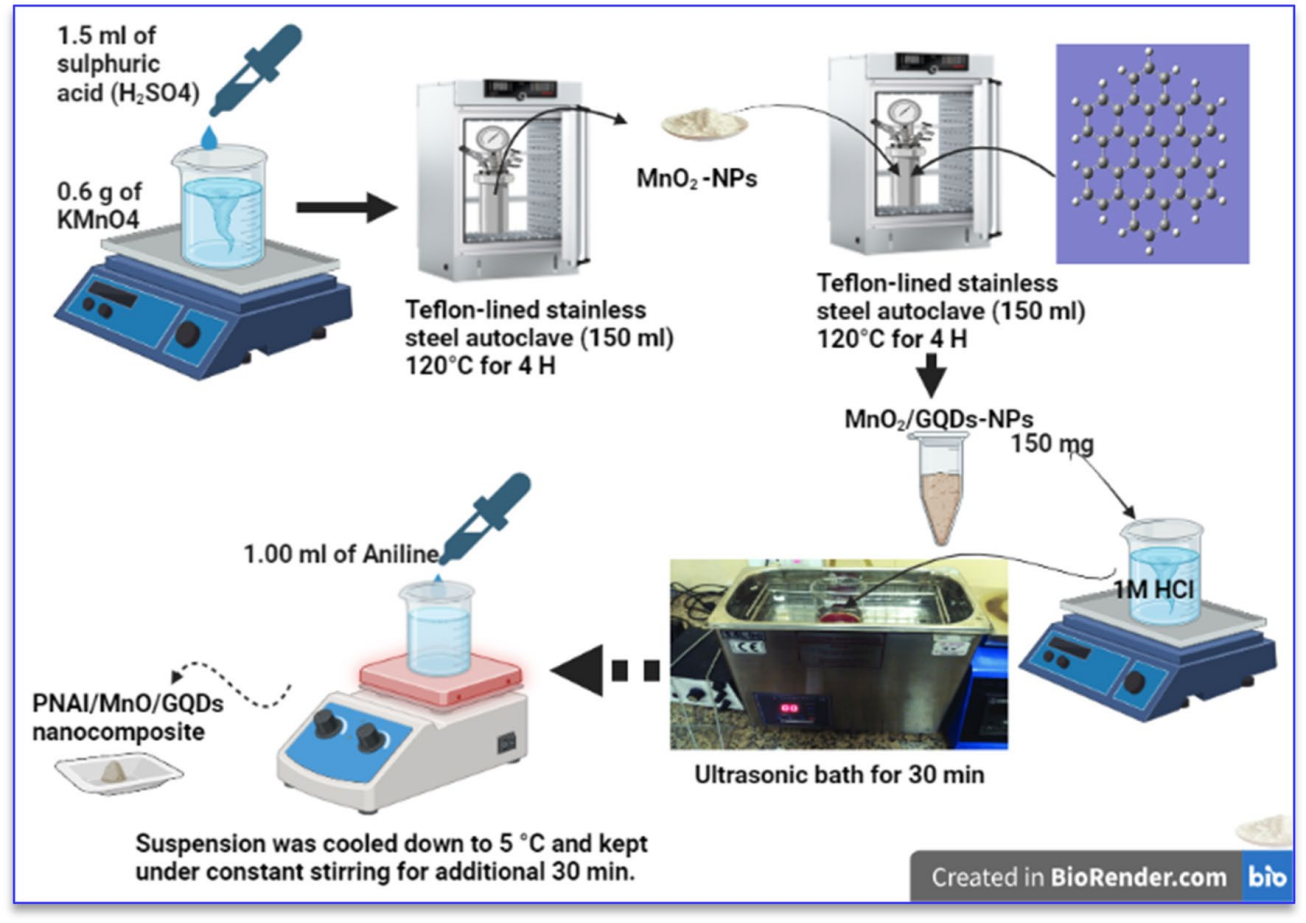
The schematic diagram for the composite synthesis process.

The ternary nanocomposite was prepared through two successive steps comprising the hydrothermal route and in-situ polymerization as represented in Fig. 1^54^.

The MnO_2_/GQDs nanocomposite was obtained via hydrothermal technique. 0.6 g of KMnO_4_ was dissolved in 45 ml of deionized Milli-Q water under continuous starring, subsequently, 1.5 ml of Conc. H_2_SO_4_ was added carefully to the dark purple solution. The solution was kept under constant stirring for an additional hour. The obtained precipitates from the solution, it was washed thoroughly and dried overnight. The PANI/MnO_2_/GQDs nanocomposite was executed through an in-situ polymerization reaction. Two separate solutions were prepared individually, then mixed. In the first beaker, 150 mg of MnO_2_/GQDs was dispersed in 45 ml of 1 M HCl aqueous solution using an ultrasonic bath for 30 min, then 1 ml of aniline was added into the suspension, it was cooled down to 5 °C and kept under constant stirring for additional 30 min. The second solution composed of 2.6 g of APS and 45 ml of 1 M HCl aqueous solution was dropped slowly to the first solution for 2 h, while the reaction temperature was kept at 5 °C. The resultant dark green precipitates were recovered, washed many times, and dried overnight. The obtained material was investigated by different characterization techniques.

### Sensor fabrication and evaluation

**Figure 2:**
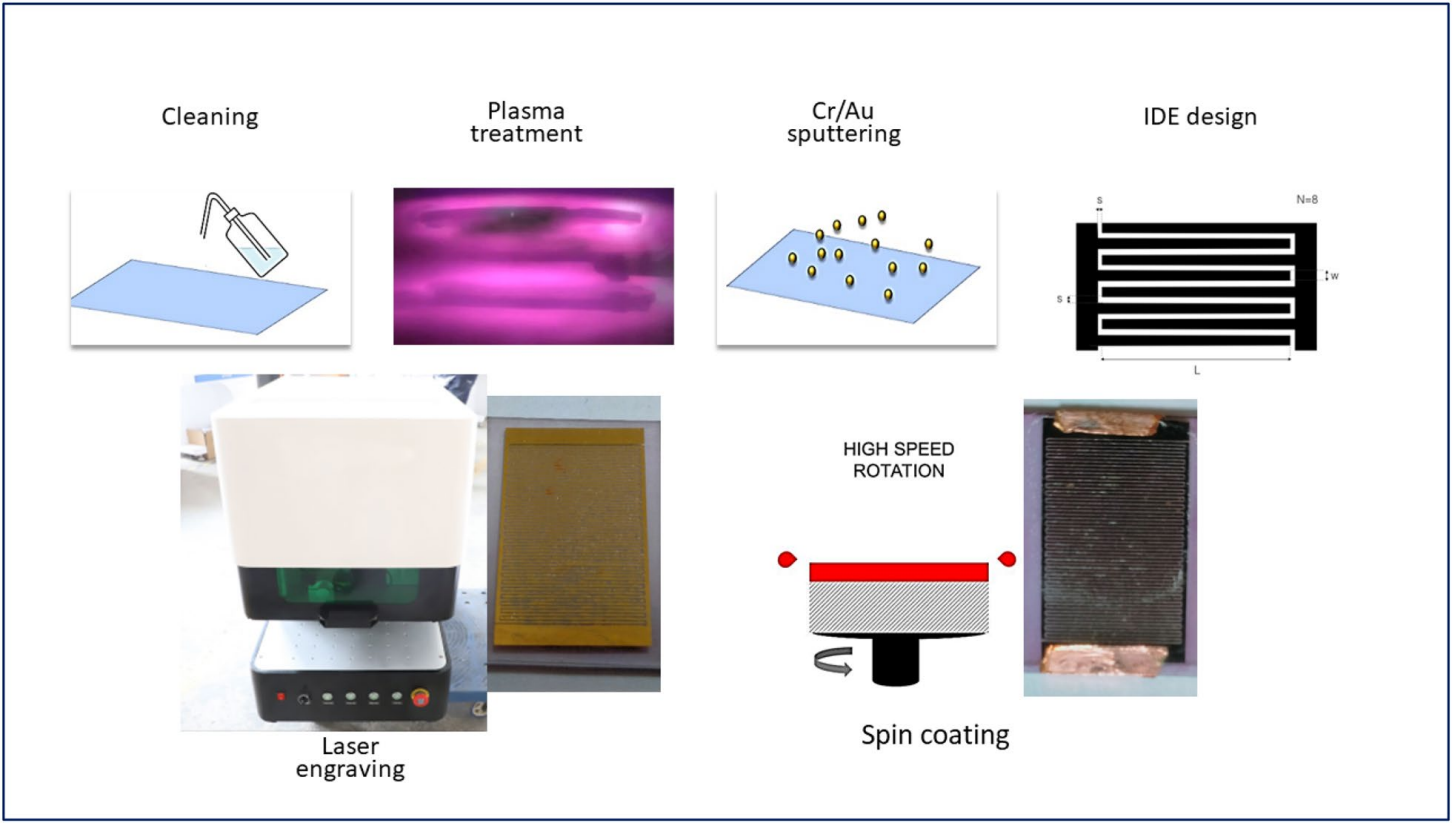
The conceptualization of the sensor fabrication.

The proposed microscopic soda–lime glass substrates were cleaned using an acidic piranha solution of 98% sulfuric acid with 30% hydrogen peroxide 3:1 to remove organic contaminations, after rinsing with DI water several times, base piranha of 3:1 mixture of Ammonium hydroxide with 30% hydrogen peroxide was applied to enhance surface quality. The slides were rinsed in type 1 DI water several times. An ultrasonic bath was used for 15 min of ethanol and acetone, respectively. Finally, the substrates were submitted to Ar plasma surface treatment to promote surface characteristics and adhesion. DC/RF sputtering VTC-600-2HD system was used for metallic layer deposition. The chamber was evacuated to a base pressure of 3 × 10^−6^ Torr then Ar (99.99%) gas for backfilling till reaching a pressure of 6 × 10^−3^ Torr as a base pressure where Ar gas flow was 20 standards cubic centimeters per minute (sccm). DC head was used to sputter a thin Cr layer of 8 nm thickness at 250° C as a wetting layer, after that, RF head was used to deposit 90 nm of Au layer at room temperature. Interdigitated electrode (IDE) patterns were fabricated using XT-Laser 50 W near-Infrared 1064 nm Q-switched Ytterbium pulsed fiber laser (Raycus RFl-P50QB) coupled with high-speed galvo scan head of 8000 mm/s maximum speed. 10 pairs of fingers of 8 mm length and 500 µm width were engraved using 20% power, 250 mm scan speed, 50 kHz frequency, and pulse duration of 13ns. The collected sensing material was mixed with Polyvinylpyrrolidone PVP 2%) and a suitable amount of de-ionized water till forming a homogeneous paste. The paste was applied on the fabricated IDE using spin coating machine at 2000 rpm. Silver paste and copper cladding were used to prepare the sensor for measurement. Figure 2 delivers the fabrication process.

**Figure 3:**
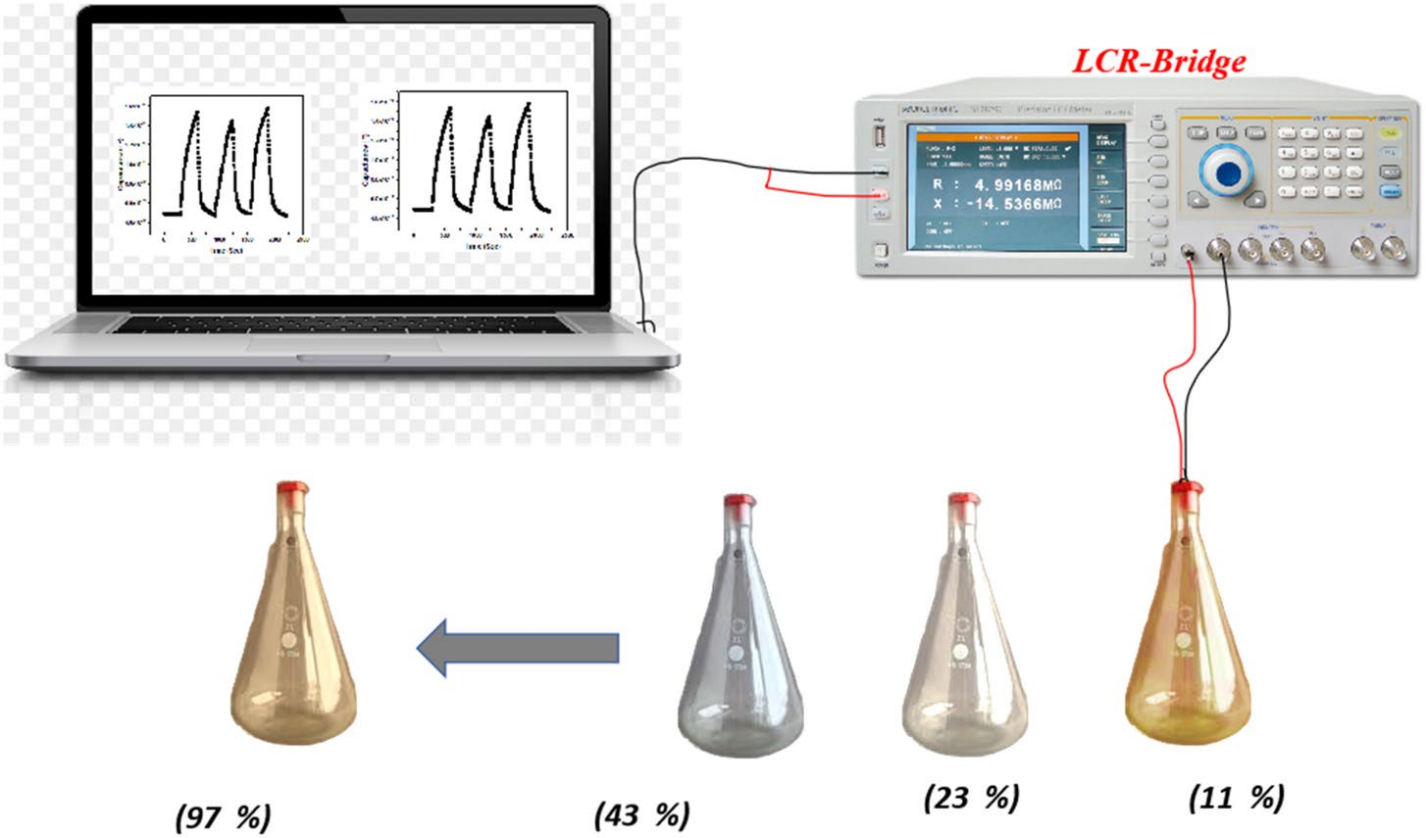
humidity sensing measurment setup.

The fabricated sensor was allowed to dry to eliminate mixing water at 60°C for 24 h. The sensor was subjected to two different levels of humidity (7% and 97%) while applying 1 VAC for 12 h to enhance its stability. The response of the sensor was evaluated using saturated salt solution in a humidity range 11%-97%. The measured parameters included sensitivity, repeatability, and response and recovery times. the sensing mechanism was explained based on complex impedance measurements up to 5 M Hz. The schematic representation of sensor fabrication is dipected in Fig. 3.

### Characterization techniques

The X-ray diffraction (XRD) planes and the pattern were investigated using the XRD Device (Malvern Panalytical Empyrean 3) within a scanning scale from 5° to 75 º, UV–Vis absorption spectra of the prepared samples were acquired using Agilent Varian Cary 5000 double-beam UV–Vis-NIR spectrophotometer in the wavelength range of 200–800 nm to study the optical properties of the prepared samples. The Fourier Transform Infrared (FTIR) was analyzed using the KBr disks method by (Vertex 70, Bruker) within a 400–4000 cm^-1^ scanning range and 4 cm^-1^ scanning resolution. Besides, Raman analysis was acquired by the device (Witec Alpha 300 RA, 514 nm excitation).

## Results and discussions

**Figure 4:**
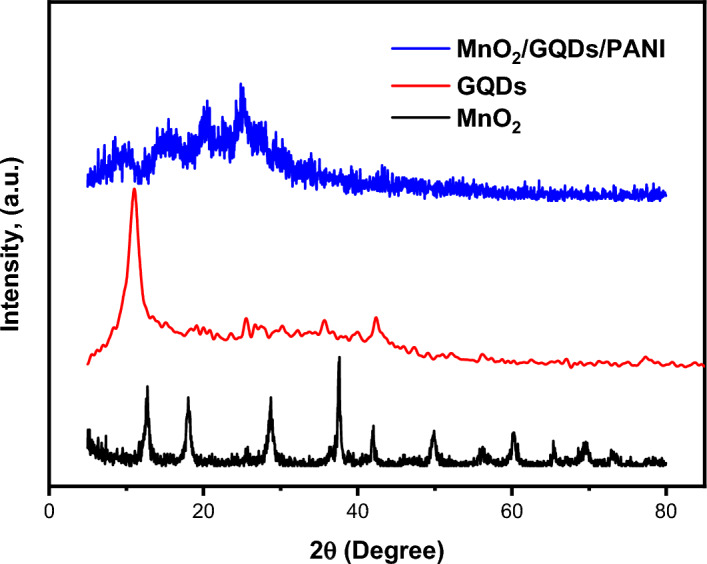
The diffraction pattern of the prepared structures.

XRD is an important technique that is considered as a primary tool for investigating the structure of various composite materials. The phase identification and composition of MnO_2_, GQDs and MnO_2_/GQDs/PANI were caried out from 2θ = 5 to 75º with a scanning rate of 2º/min as demonstrated in Fig. 4. The apparent diffraction planes of the α-MnO_2_ were observed. The tetragonal system of the MnO_2_ with a space group of I4/m was verified. The diffraction planes were observed at the angles of 12.8°, 18.07°, 28.7°, 37.55°, 41.96°, 49.9°, 56.3°, 60.2°, 65.4°, and 69.5°, corresponding to the planes of (110), (200), (310). (211), (301), (411), (600), (521), (002), and (541), respectively, with the most intense peak residing at a degree around 37 º. The results are in acceptable agreement with prior reported works^55,56^. For the composite, the three components' card numbers and peaks are included as 00–012-0720, 00–048-1206, 00–065-0826 for MnO_2_, Carbon component, and the PANI, respectively.

The corresponding card no. is PDF 01–072-1982. While implementing other constitutes of the structure, the material's sharpness and the intensity of the peaks get a bit broader and become distorted. This might be attributed to the occurring rearrangement of the structure for the atoms, resulting in a deformation of the composite structure. The nominal crystallite size of the structure is calculated based on the Scherrer equation as previously reported^57^.

1 $$D \left(crystallite\, size\right)=\left\{\frac{k\lambda }{\beta Cos \theta }\right\}$$

where, β represents the full width at the midpoint of the maximum value of the peak, the diffraction angle is defined as theta (θ), K is the shape factor and λ the incident wavelength of the copper source. The resulting degree of crystallinity is calculated from the diffraction patterns as the ratio between the area under crystalline and non-crystalline peaks as^27^

2 $$Xc=\left(\frac{Area(c)}{Area (C+A)}\right)\times 100$$

Area (c), Area (A) are the crystalline and amorphous areas respectively.

The dislocation density existing is determined from the inverse of the crystallite size as

3 $$\delta ={D}^{-2}$$

The values of crystallite size (D), crystallinity degree (XC), and dislocation density (δ) are 20, 15 nm and 75%, 36%, and 25 × 10^–4^, 44 × 10^–4^ for MnO_2_ and MnO2-GQDs-PANI composite respectively.

**Figure 5:**
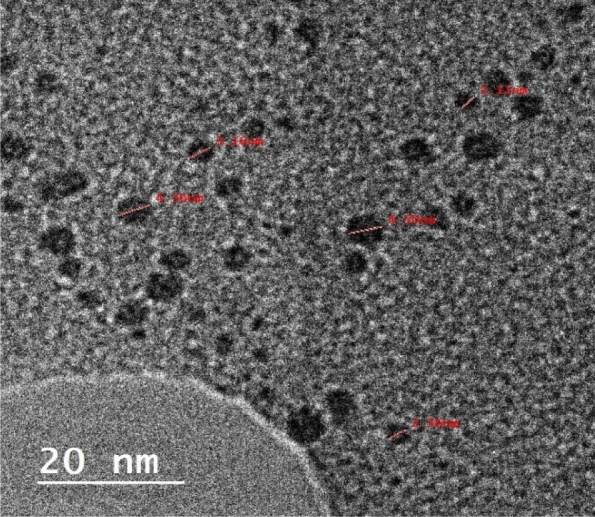
The HRTEM image of the GQDs.

The HRTEM of the GQDs is illustrated in Fig. 5. The HR-TEM image demonstrates that the GQDS have sphyrical shape. The average particle size as measured from the provided image is about 5 nm on average. The GQDs seems to be separated, it reveals no agglomeration.

**Figure 6:**
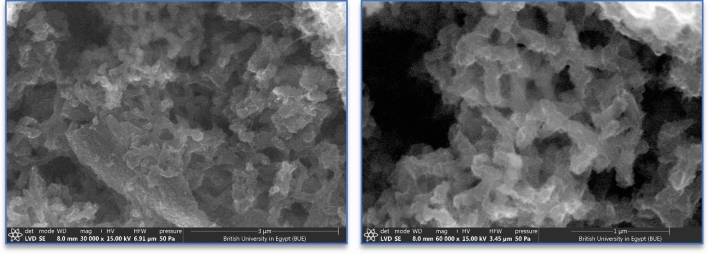
The SEM of the prepared GQDs-MnO_2_-PANI structure with different magnifications.

The surface morphological features of the MnO_2_/GQDs/PANI were examined by scanning electron microscope (SEM) at two different magnifications as shown in Fig. 6.

**Figure 7:**
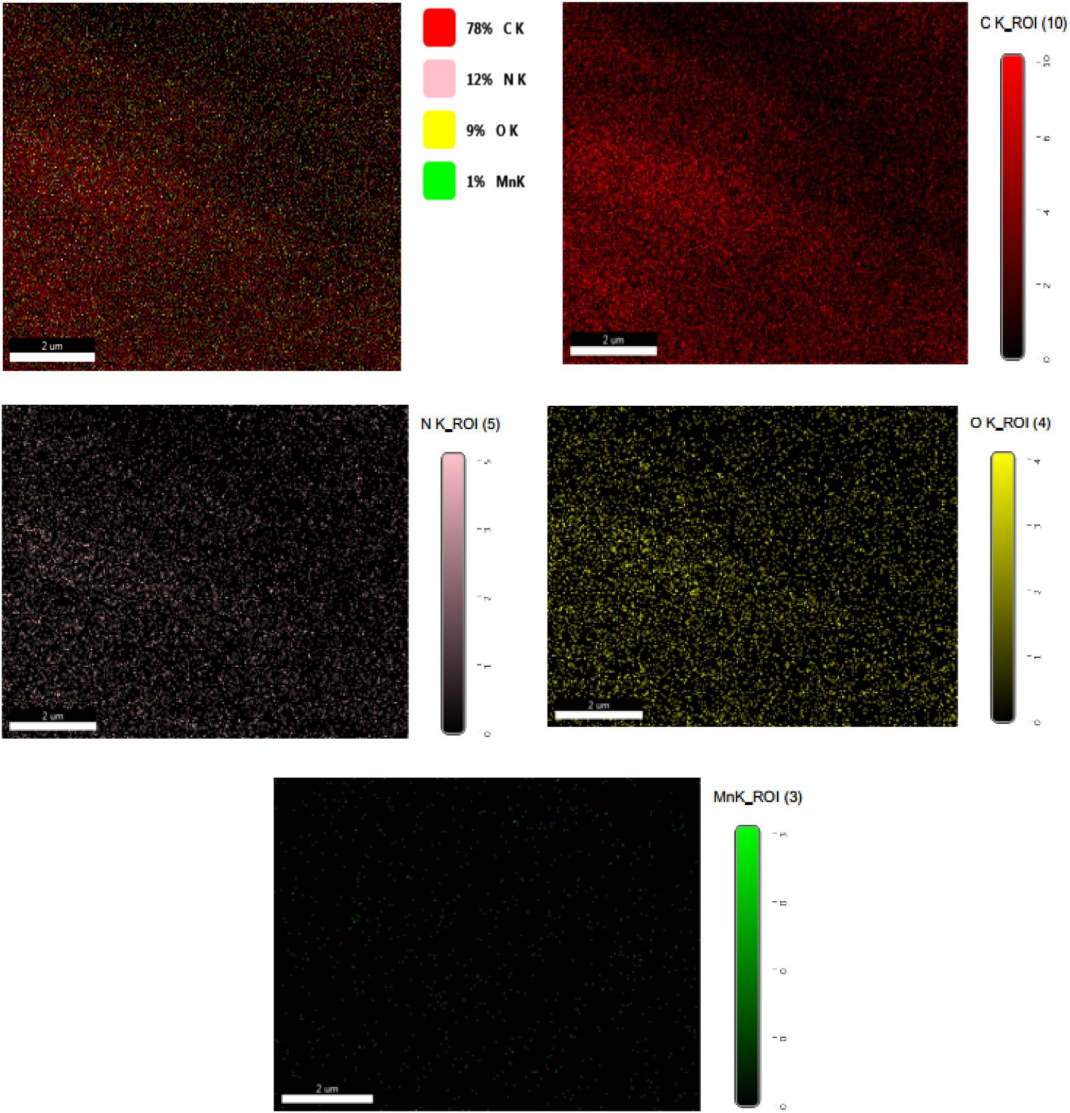
The elemental mapping of the MnO_2_/GQDs/PANI ternary composite.

The low magnification SEM image demonstrates a fibrous-like structure of PANI. The entire sample just reveals the presence of PANI without any evidence of the presence of other materials. By looking at the higher magnified SEM image, the fibers are clearly demonstrated with some other irregular ferments. The PANI could have covered the entire MnO_2_ particles, thereby no evidence for the presence of these particles (MnO_2_). More importantly, the dimensions of GQDs are too small to be seen by this technique. the XRD can penetrate through the sample to some specific depth, hence the presence of MnO_2_ particles was confirmed through XRD measurements. By combining the results of both techniques (XRD and SEM), it was concluded that the MnO_2_ nanoparticles and GQDs are present and covered with the PANI fibers. The elemental mapping of the ternary composite is shown in Fig. 7.

The elemental mapping of the ternary composite MnO_2_/GQDs/PANI prove the presence of oxygen carbon and nitrogen as a proof fro the presence of PANI and GQDs. The elemental mapping analysis proves the presence manganese as a constuent elemnt of the ternary nano composite. the distribution of the individual elements seems to homogeniuos without any agglomeration.

**Figure 8:**
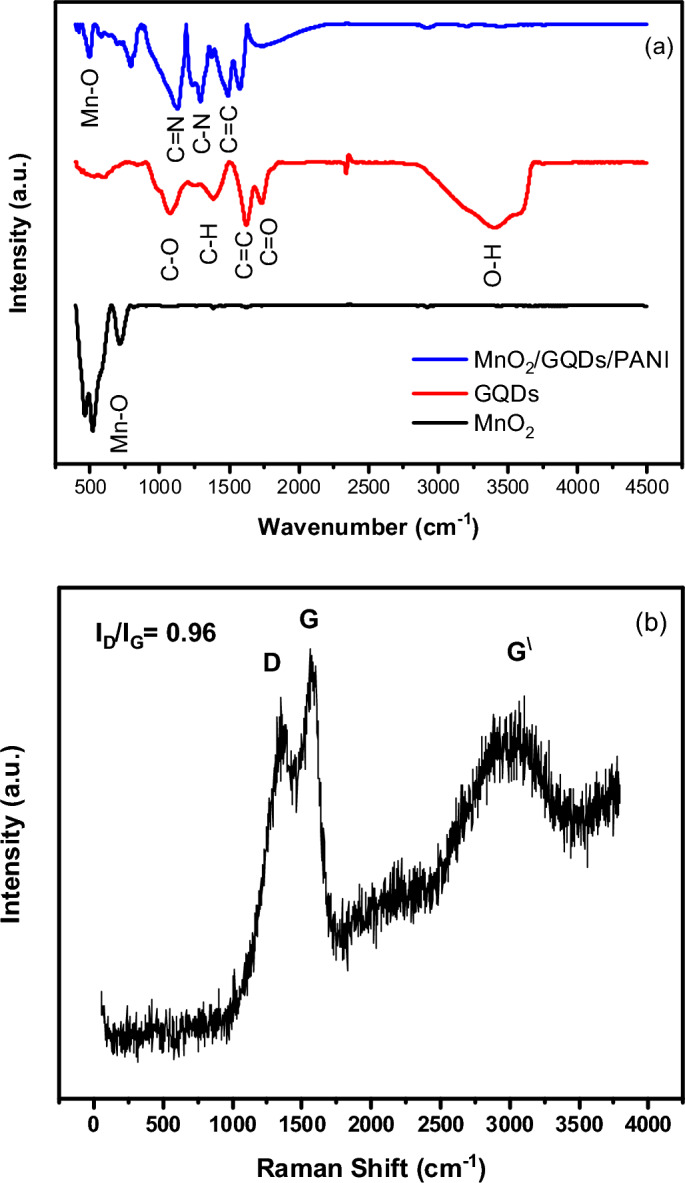
(**a**) The FTIR of the prepared GQDs-MnO_2_-PANI compared with GQDs, and PANI structures and (**b**) the Raman spectra of the structure (GQDs-MnO_2_-PANI).

The chemical bonding and interaction between elements were verified through the FTIR measurements from 4000 cm^−1^ to 400 cm^−1^ with a spectral resolution 4 cm^-1^. The FTIR spectra of MnO_2_, GQDs and nanocomposite (MnO_2_/GQDs/PANI) are depicted in Fig. 8a. The absorption peaks of GQDs at 1062 cm^−1^, 1381 cm^−1^, 1611 cm^−1^, and 1738 cm^−1^ are ascribed to the C–O stretching vibrations, C–H bindings, C = C, and C = O, respectively. The FTIR spectrum of MnO_2_ reveals an absorption band at 710 cm^−1^ that arises from the stretching vibrations of the Mn–O. The nanocomposite (MnO_2_/GQDs/PANI) demonstrates the characteristics band of MnO_2_, GQDs and PANI. The benzene ring presents a stretching vibration at 1487 cm^-1^ due to the presence of C = C^58–60^.

The Raman spectrum of the composite nanostructure is presented in Fig. 8b. Raman analysis has a fingerprint spectrum regarding the carbon nanomaterials, where the locations of the bands beside its relative intensity used to distinguish between different nanocarbon derivatives. The three main bands characterizing all carbon nano-based materials are D, G and G^\^. The disordered band (D band at 1339 cm^−1^) is related to the defects that originate from the carbon atom vibrations with dangling bonds. G band at 1564 cm^−1^, the vibration of carbon atom in the hexagonal lattice due to the E_2g_ phonon mode of sp^2^ is resulted in the graphitization band. More additional wide band at 3022cm^−1^ is devoted to G^\^ or overtone. One of the most valuable parameters that are generally used to judge the quality and the intercalation of the prepared materials is I_D_/I_G_ ratio. The intercalation between GQDs and PANI can be verified by measuring the I_D_/I_G_ ratio. This ratio was estimated and found to 0.96, thus indicating significant defects, that promote the sensitivity of the materials to humidity measurement^61^.

**Figure 9:**
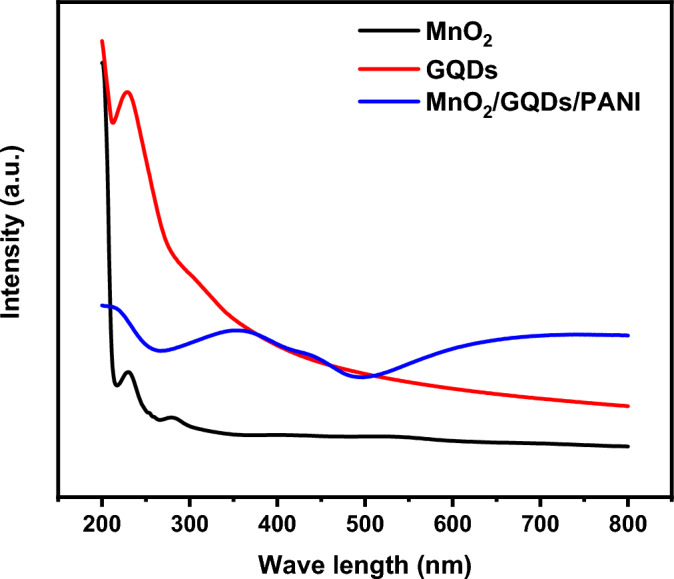
UV–Vis spectra of the prepared structures.

MnO_2_/GQDs/PANI is recorded from 200 cm^−1^ to 800 cm^−1^ as displayed in Fig. 9. GQDs exhibit an absorption peak at 212 nm related to the π–π^*^ transition due to the C = C bonds. The absorption band at about 281 nm is due to the electronic transition of the pure MnO_2_ nanoparticles. The electronic transition of π → polaron for PANI demonstrates a characteristic peak at about 485 nm. The most surprising phenomenon is the formation of radical cations in PANI that improves the delocalization of charges in PANI thereby enhancing the electrical conductivity of the PANI. the characteristic peak of PANI is in the range 400 -430 nm, however, the absence of this band, blue shift in the UV spectra, and the presence of a broad peak, confirm the incorporation of MnO_2_ and PANI conducting matrix during the *in-situ* polymerization process^62^.

**Figure 10:**
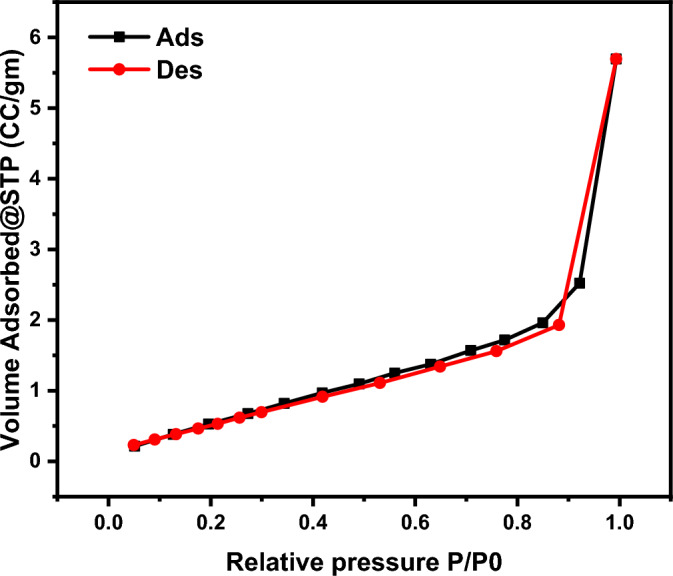
BET surface area of MnO2/GQDs/PANI.

The N_2_ adsorption–desorption isotherm of the ternary MnO_2_/GQDs/PANI is illustrated in Fig. 10. According to the International Union of Pure and Applied Chemistry (IUPAC) classification, the N_2_ adsorption–desorption isotherm is belong to type III. In this case (type III isotherm), the interaction between the adsorbent-adsorbate are relatively weak, and the adsorbed molecules are accumulated all over the active sites on the surface of the studied material. The BET surface area of the ternary MnO_2_/GQDs/PANI was found to be 54.9 m^2^/g. Different parameters have been measured and calculated from N_2_ adsorption–desorption isotherm and tabulated in Table 1.

**Table 1:** Complete N_2_ adsorption–desorption isotherm data for MnO_2_/GQDs/PANI composite.

Average Pore Size, nm	BET surface area, m^2^/g	BJH surface area, m^2^/g	BJH Pore Volume, cm^3^/g	Pore radius, nm	DFT surface area, m^2^/g	DFT pore volume, cm^3^/g	Total Pore Volume
6.8501	54.9	32.32	0.174727	1.933	46.35	0.1752	0.18792

### Humidity sensing measurements

The humidity sensing experiments were carried out using a saturated salt solution to generate a fixed relative humidity level. The saturated salt solutions were stored in a closed vessel to generate the required humidity level. The investigated sensor was inserted above the saturated salt solution in the closed vessel for a while, then the impedance/capacitance change was recorded using LCR meter at different frequencies (50 Hz, 200 Hz, 500 Hz and 1kHz). The sensor was evaluated in a humidity range of 11–97%, at different frequencies from 50 Hz up to 100 kHz. All experiments were conducted at room temperature. In these measurements, the impedance and capacitance variations were recorded as a function of humidity level to evaluate the humidity sensing performance of the studied structure.

**Figure 11:**
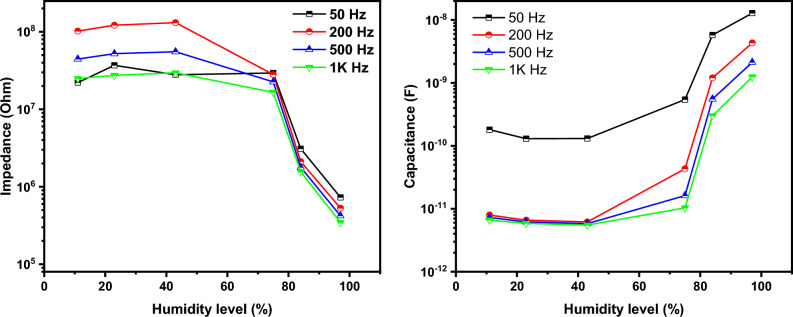
The alteration of the impedance magnitude with humidity levels at various frequencies.

The impedance/capacitance variation as a function of relative humidity is displayed in Fig. 11. It is apparent that the impedance/capacitance variation depends on the testing frequency. This is a common characteristic for all humidity sensors and related to the polarizability of water molecules. As the frequency increases, the adsorbed water molecule cannot trace the alternation of the applied AC signal^18^. Based on the measured values for impedance and capacitance, the optimal testing frequency was found to be 200 Hz. The impedance decreases when the capacitance increases as the humidity increases. The measured curves can be divided into two regions, low humidity up to 43% and high humidity from 75% up to 97%. In the first region the impedance/capacitance variation is less than that of the second region. The impedance variation seems to be linear at high humidity level (greater than 75%). The capacitance variation is mostly having an expositional increment trend. The sensitivity can be defined as the change of measured signal per change in relative humidity level. the sensitivity can be estimated using the following Eqs. ^17,54,63–65^:

4 $${S}_{Z}= \frac{{Z}_{11 }- {Z}_{H}}{{RH}_{H }- {RH}_{11}}\mathrm{ for\, impedance}$$

5 $${S}_{c}= \frac{{C}_{H }- {C}_{11}}{{RH}_{H }- {RH}_{11}}\mathrm{ for\, capacitance}$$

The calculated sensitivities are 1.18 × 10^6^ Ω/RH and 5.02 × 10^−11^ F/RH for impedance and capacitance measurements respectively.

**Figure 12:**
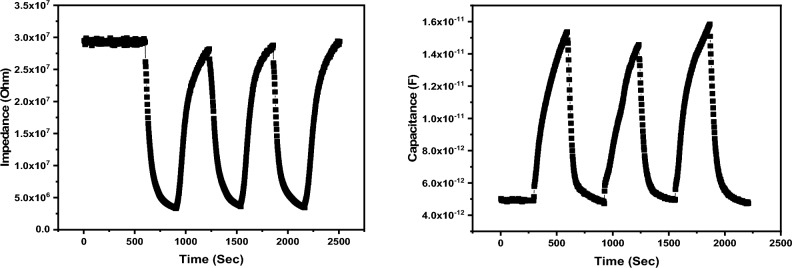
The study of the sensor stability showing the repeated cycles performance.

**Figure 13:**
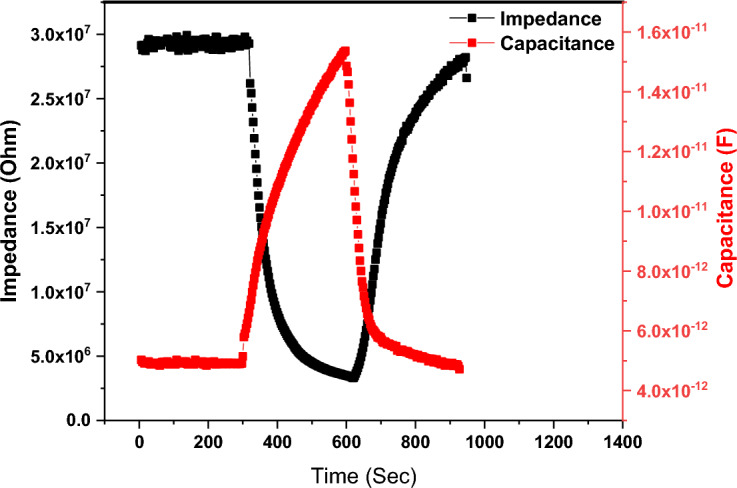
The response and recovery times of the studied sensor.

The repeatability of the sensor describes the ability of the sensor to offer the same value of change when subject to two different humidity levels many times. The repeatability could be a good way to describe the stability of the sensor over a short period of time. The repeatability of the sensor was tested for three successive cycles between 11 and 43%. The repeatability of the sensors is described in Fig. 12 where the change in the impedance and capacitance was recorded every 3 s. It was noticed that the tested sensor exhibits a good repeatability with approximately no drift. The impedance of the tested sensor has approximately the same value, while the capacitance changed slightly as the humidity changed. However, the overall regime of the impedance and capacitance variations are satisfactory. The obtained results indicate an excellent repeatability of the tested sensor. The response and recovery times are one of the most significant values that determine the rate of the response. When a humidity sensor is subject to a controlled humidity level, it requires a specific time to respond, known as response time. On the other hand, the recovery time is the time taken by the sensor to attain its original (base line) value. The response and recovery times of the studied sensor is demonstrated in Fig. 13. The response and recovery behavior of the sensor were found to be reproducible; it responds to humidity increment then return to its base line value at low humidity condition. As affirmed earlier, the impedance decreases, and capacitance increases as the humidity level increases. When compared to each other, the impedance variation is more uniform than capacitance variation. The impedance curve shows approximately no drift, while a small drift in the second cycle of capacitance variation curve is recognized.

To get a closer vision, response and recovery time were studied as depicted in Fig. 7. The response and recovery times were estimated and found to be 170 s and 229 s. However, the response and recovery times are long, the studied sensor exhibited a reliable change in its impedance and capacitance.

The humidity sensing mechanism can be explained based on complex impedance spectroscopy (CIS) measurements.

**Figure 14:**
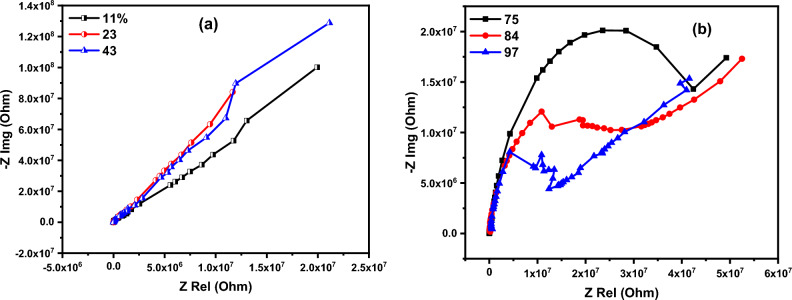
The sensing mechanism using Cole–Cole diagram.

The sensing mechanism can be divided into two regimes. The first regime comprises low humidity up to 43%, whereas the relationship between real and imaginary parts of impedance is linear as depicted in Fig. 14a. The second regime includes high humidity up to 97% as shown in Fig. 14b, which is characterized by a short tail connected to a semicircle. The shape and shape of the impedance curves are indicators for the sensing mechanism. The adsorbed water molecules can be visualized as sequential layers that bonded to the surface of the sensor. At low humidity conditions, the adsorbed water molecules (first adsorbed layer) are bonded to the surface of the sensor via a double hydrogen bond. This case inhibits the movement of charge carriers; hence the sensor cannot respond to the presence of humidity. As the humidity increases, more water molecules are adsorbed over the first adsorbed water layers. In this case, the generated hydronium ions act as the main charge charier, where the protons can jump freely among adjacent water molecules. The presence of polyaniline in the studied structure is the reason for the production of hydronium ions (H_3_O). The synthesized emeraldine base polyaniline has two forms of oxidized (−N =) and reduced (_—_NH_—_) groups. The presence of un-bonded electron pair on the nitrogen atom resulted in the protonation of above stated forms (–N = and _—_NH_—_). This can be described as follows:

6 $${\text{NH }} \to {\text{ NH}}_{{2}}^{ + } {\text{and N }} \to {\text{ NH}}^{ + }$$

The conduction mechanism of the polyaniline is governed by the electron hopping from protonated reduced form (NH_2_^+^) to the protonated oxidized form (NH^+^). Since (NH_2_^+^) is incapable to leave an electron without earlier leaving a proton, thus the electron hopping occurs conditionally before the transformation of a proton. This type of conduction occurs in the presence of water molecules^66^.

7 $${\text{NH}}_{{2}}^{ + } + {\text{H}}_{{2}} {\text{O}} \to {\text{NH}} + {\text{H}}_{{3}} {\text{O}}^{ + }$$

**Figure 15:**
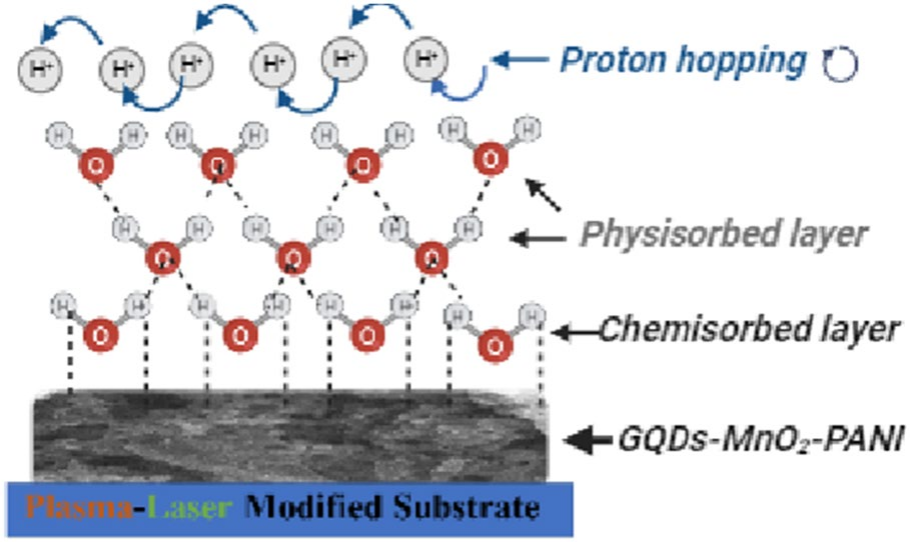
The schematic diagram of the humidity sensing mechanism of the studied structure.

The proposed sensing mechanism is provided schematically in Fig. 15.

The results of the obtained humidty sensor was comapered with thous obtained from similar structures as shown in Table 2.

**Table 2:** The compoarison between the proposed structure and close structures for humidity sensing application.

Structure	Response/recovery times	Humidity sensing range	References
GQDs	5 s/NA	15–80% RH	^67^
N-S co-doped graphene quantum dots (GQDs)	15 s/55 s	40–90% RH	^49^
(RGO)/poly(diallylimethyammonium chloride)	94 s/133 s	11–97% RH	^68^
Graphene quantum dots prepared via carbonization of citric acid	10 s/NA	0–97% RH	^69^
Ag nanoparticles decorated with graphene quantum dots	25 s/30 s	25–95% RH	^50^
Polyaniline/Water soluble graphene oxide	8 s/9 s	11–97% RH	^3^
polyaniline-Yttrium oxide composite	3 s/4 s	11–97% RH	^1^
Polyaniline	180 s/ 60 s	10–97% RH	^9^
MnO_2_/GQDs and PANI	120 s/220 s	11–97% RH	This work

## Conclusion

In the studied work, ternary composite of polymeric matrix as polyaniline combined with graphene quantum dots and MnO_2_. The validity of the preparation strategy is explored via versatile examination apparatus such as XRD, SEM, FTIR, UV–Vis, and Raman spectra. The dimensions of the crystallite size and other related structural parameters are emerged from the XRD, and SEM which verifies the nanoscale and crystallinity of the structure. The feasible and resulted implantation of the components is promoted through the bands arising in FTIR, and Raman as well as UV–Vis spectra. The structure is investigated for the humidity sensing throughout a wide scale of humidity levels up to 97 RH% which verifies the decent response and recovery times in both capacitance and impedance modes. The delivered structure might pursue a desirable set of specifications directed towards humidity sensing for the upcoming years in various areas.

## Data Availability

All data regarding this work is included in the manuscript.
